# Up-regulated oncoprotein P28GANK correlates with proliferation and poor prognosis of human glioma

**DOI:** 10.1186/1477-7819-10-169

**Published:** 2012-08-22

**Authors:** Yang Yang, Chunli Zhang, Li Li, Yusong Gao, Xinming Luo, Yadong Zhang, Weiping Liu, Zhou Fei

**Affiliations:** 1 Department of Neurosurgery, Xijing Hospital, The Fourth Military Medical University, 17 West Changle Road, Xi'an, 710032, China; 2 Department of Neurosurgery, The Zhumadian city center Hospital, 747 Zhonghua Road, Zhumadian, 463000, China; 3 Department of Pathology, Affiliated Hospital of Yan’an University, Yan’an, 716000, China; 4 Department of Neurosurgery, The 159th Hospital of PLA, 1 Fengguang Road, Zhumadian, 463008, China

**Keywords:** p28GANK, Glioma, Prognosis, siRNA

## Abstract

**Background:**

The significance of p28GANK in gliomas remains unknown. This study aims to clarify the clinical significance of p28GANK in human gliomas.

**Methods:**

The expression of p28GANK in 138 gliomas and 50 matched para-cancerous tissues was detected by immunohistochemical staining, and statistical analyses were performed to test the correlation of p28GANK with clinical parameters. To investigate the effects of p28GANK down-regulation on the growth of cells both *in vitro* and *in vivo*, an siRNA targeting p28GANK was transfected into U251 cells.

**Results:**

P28GANK expression was significantly higher in tumor specimens than in matched para-cancerous tissues. Over-expressed p28GANK significantly correlated with high karnofsky performance score (KPS), advanced WHO grade and poor overall survival of the patients. Univariate analysis showed that WHO grade and KPS also correlated with the survival of patients, and multivariate analysis suggested that KPS and p28GANK expression were two independent prognostic factors. Moreover, p28GANK gene silencing decreased the malignant growth of U251 cells both *in vitro* and *in vivo.*

**Conclusions:**

Increased expression of p28GANK is correlated with poor clinical outcomes in glioma patients. The down-regulation of p28GANK significantly inhibited cell proliferation, indicating that p28GANK might be a potential therapeutic target for glioma treatment.

## Background

Human gliomas are the most common primary intracranial tumor, accounting for the most aggressive and malignant phenotypes and resulting in poor clinical outcomes [1]. Glioma cells characteristically possess high proliferation and invasion potentials, which explain their aggressive phenotype [2]. Several oncoproteins were identified to be involved in the malignant development of human glioma [3-5]. Despite advancement in surgery and other treatments, the postoperative prognosis of glioma patients is still unsatisfactory. Therefore, the identification of novel markers for gliomas is critical for the improvement of therapeutic strategies and the individualization of therapeutic interventions.

P28GANK, also named as PSMD10 and gankyrin, is a highly conserved protein with six ankyrin repeats in mammals [6,7], which is located on human chromosome Xq22.3 and cloned by complementary DNA subtractive hybridization in hepatocellular carcinoma (HCC) [8]. Previous studies revealed that p28GANK promotes liver regeneration in patients with hepatic failure by regulating cell cycle progression of normal hepatocytes [9]. P28GANK is widely over-expressed in HCC compared to normal hepatic tissues [10], and plays critical roles in the progression, invasiveness and metastasis of HCC [11,12]. The upregulation of p28GANK expression correlated with poor clinical outcomes in human esophageal squamous cell carcinoma [13], colorectal cancer [14], pancreatic cancer [15] and lung cancer [16], indicating that p28GANK is an important oncoprotein involved in the carcinogenesis of various human cancers. However, the significance of p28GANK in the malignant progression of gliomas is still unknown.

In the present study, to investigate the importance of the p28GANK in gliomas, immunohistochemisty was performed to detect p28GANK expression in 138 gliomas and 50 matched para-cancerous tissues. The relationships between p28GANK expression and clinical factors, including age, gender, Karnofsky performance status (KPS), World Health Organisation (WHO) classification and prognosis in tumors, were also analyzed. Then, lentivirus-mediated siRNA that targeted p28GANK was transfected into a cell line that highly expressed p28GANK, to investigate the effects of p28GANK downregulation on the growth of gliomas both *in vitro* and *in vivo.*

## Methods

### Samples and cell line

A total of 138 glioma tissues and 50 matched para-cancerous tissues were obtained from patients, including 59 men and 79 women ranging from 7 to 69 years old (mean ± SD, 42.3 ± 14.8), who had received tumor resection between January 2004 and August 2006, at the Department of Neurosurgery, Xijing Hospital, Fourth Military Medical University, Xi’an, China. None of the patients had received preoperative anti-tumor treatment. Clinical features including age, gender, KPS and WHO classification were obtained from the medical records. Among these patients, 82 had high-grade gliomas (grade III + IV), and 56 patients had low-grade gliomas (grade I + II) according the 2000 WHO criteria. With regard to KPS, the patients were divided into two groups, each containing 69 patients, either above or below the median score (70). All of the patients consented to the use of resected samples, and written informed consent was also obtained. The present study was approved by the Hospital’s Protection of Human Subjects Committee. For survival analysis, five-year follow-ups were executed by telephone or written correspondence. Patients who died due to causes unrelated to tumors or without a complete follow-up prior to death were excluded from the present study.

The human glioblastoma cell line U251 was purchased from American Type Culture Collection and maintained in our lab. The cells were cultured in DMEM (Life Technologies, Inc., Grand Island, NY, USA) with 10% fetal bovine serum (Life Technologies, Inc.) at 37°C in a 5% CO2 incubator.

### Immunohistochemical staining

Immunohistochemical staining was performed to detect p28GANK expression in surgical specimens from glioma patients. After deparaffinisation and blocking, the sections were incubated with PBS diluted p28GANK antibody (Abcam, 1:300) overnight. After three 5-minute washes, the sections were incubated with biotinylated goat anti-Rb IgG/HRP for 2 h. Following three additional 5-minute washes with PBS-T, DAB solution was used for the visualization. Negative controls were executed by replacing the primary antibody with PBS. The results were determined independently by two highly-trained pathological experts, following the standards of a previous study [17]. Briefly, the intensity of each side was scored as 0, 1, 2, or 3, and the proportion was scored 0 (0%), 1 (0 to 30%), 2 (30 to 60%) and 3 (> 60%) respectively. The final results were the addition of two scores: approximately 0 to 2 negative (−) and 3 to 6 positive (+) expression.

### Western blot

Western blot assays were performed as described in a previous study [14]. Total protein was separated by sodium dodecyl sulphate–polyacrylamide gel electrophoresis and transferred onto an NC membrane (Bio-Rad, Hercules, CA, USA). After blocking with 10% defatted milk in PBS, the membrane was incubated with rabbit anti-p28GANK (Abcam, 1:500) in 2% defatted milk. The p28GANK protein level was detected using the ECL reagents (Pierce, Rockford, IL, USA). β-actin was used as a loading control.

### Lentivirus-mediated siRNA construction and transfection

The sequence of siRNA for p28GANK was 5′-CTGACCAGGACAGCAGAAC-3′, and the scrambled sequence (5′-CCAGAAGAGCAATCTGTAC-3′) that did not target the known gene was used as a negative control. The sequences were synthesized and inserted into a pGCSIL-GFP vector by Genechen Co, LTD, Shanghai, China. The vectors were transfected into U251 cells, and the green fluorescent protein (GFP)-positive cells were purified by flow cytometry (FACScan; Becton Dickinson, San Jose, CA, USA), and named Si- U251 and Con-U251.

### MTT assay

#### MTT is the abbreviation of 3-(4,5-dimethylthiazol-2-yl) -2,5 -diphenyl-tetrazolium bromide

An MTT assay was used to evaluate cell proliferative ability as described previously [14]. After being cultured for 1, 2, 3, 4, 5, 6 and 7 days, MTT solution (5 mg/ml; Sigma, St. Louis, MO, USA) was added to each well for 4 h. Then, the supernatant was replaced with 150 μl dimethyl sulfoxide (DMSO) to dissolve the crystals by agitation for 10 minutes. The absorbance values were evaluated by an ELISA reader (Bio-Rad Laboratories, Richmond, CA, USA) at a wavelength of 490 nm. Each experiment was repeated in triplicate, and the result shown is the mean of three replicates.

### Nude mouse experiments

BALB/c nu/nu mice were used for subcutaneous tumorigenesis experiments following the guidelines of the NIH Animal Care and Use Committee. The siRNA- transfected cells were injected into the left flanks of six mice, while control cells were injected into their right flanks. After inoculation for 30 days, the mice were sacrificed, and tumor volumes were determined by the formula of 0.5 × length × width^2^.

### Statistical analysis

Statistical analyses were performed using the SPSS 17.0 software package (SPSS, Chicago, IL, USA), and the value of *P* < 0.05 was assigned to be statistically significant. The Wilcoxon rank sum test was performed to analyze the differential expression of p28GANK in tumor and matched para-cancerous tissues. The Kruskal-Wallis *H* test was used to test correlation between p28GANK expression and various clinical parameters, followed by one-way analysis of variance (ANOVA) for the evaluation of the difference between three comparisons in cell proliferation. A least significant difference *t-*test was used for the analysis of animal experiments. Then, the Kaplan-Meier method was performed to plot overall survival curves, followed by the log-rank test for statistical significance, and Cox multivariate regression analysis.

## Results

### Increased expression of p28GANK in gliomas

**Figure 1:**
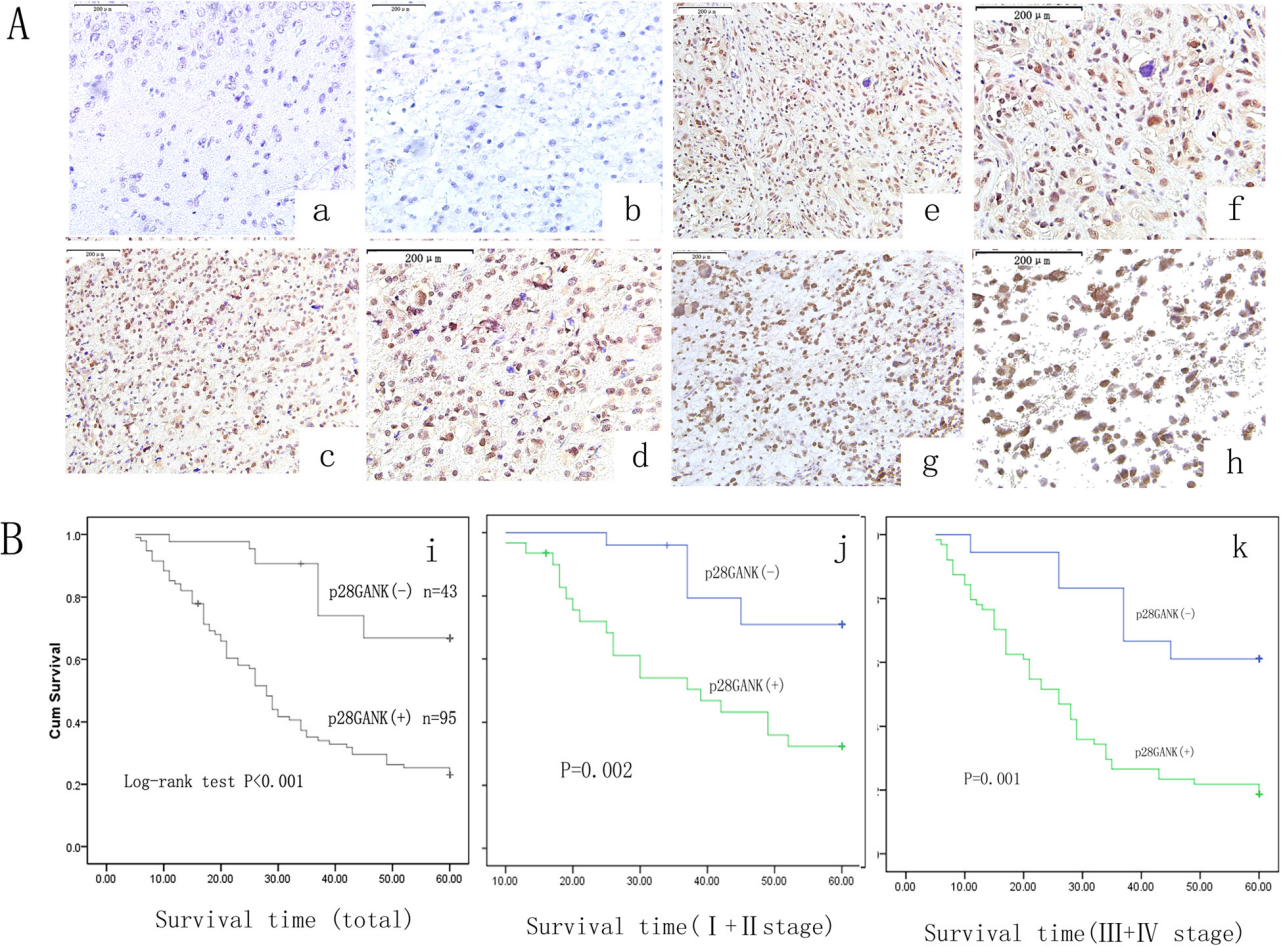
**Immunohistochemical staining of glioma and para-cancerous tissues (A) and Kaplan-Meier analysis of the p28GANK expression associated with the survival time of patients after surgery(B).** (**A**) Staining of p28GANK was observed mainly in the nucleus of glioma cells. **a** Negative staining (−) of p28GANK in para-cancerous tissues. **b** Absent staining (−) of p28GANK in glioma cells. **c, d** Positive staining (+) of p28GANK in grade II glioma cells. **e, f** Positive staining (+) of p28GANK in grade III glioma cells. **g, h** Positive staining (+) p28GANK in grade IV glioma cells. Original magnifications: **a**-**c**, **e** and **g** × 200; **d**, **f**, **h** × 400. (**B**) Survival rates of total (**i**), gradeI + II (**j**) and grade III + IV (**k**) patients according to the expression of p28GANK.

Immunohistochemical staining was performed to evaluate p28GANK expression in 138 glioma and 50 matched para-cancerous tissues. Positive p28GANK staining was observed in the nucleus and/or cytoplasm of tumor cells, which exhibited positive staining in 95 tumor samples and 8 para-cancerous tissues (Figure 1A). Statistical analysis showed that the positive staining rate in glioma tissues (68.9%) was significantly higher than in matched para-cancerous tissues (16%) (*P* < 0.01).

### Enhanced expression of p28GANK correlated with clinical features of glioma patients

Positive p28GANK staining was observed in 95 samples (68.9%), and 43 samples (31.1%) were negative for staining. Statistical analyses showed that p28GANK expression was significantly higher in advanced-grade tissues than in low-grade tissues (*P* = 0.005). In addition, a higher p28GANK expression was significantly associated with a high KPS (*P* < 0.001). In contrast, there was no statistically significant difference in p28GANK expression with regard to age and gender factors (*P* > 0.05) (Table 1).

**Table 1:** Association of p28GANK expression with the clinical parameters

**Feature**	**Total**	**p28GANK expression**		** *P* -value**^**a**^
		**Negative**	**Positive**	
Gender				
Female	79	21	58	0.18
Male	59	22	37	
Age, years				
< 42	69	20	49	0.58
> 42	69	23	46	
KPS, score				
< 70	69	38	31	< 0.001
> 70	69	5	64	
Grade				
I + II	56	25	31	0.005
III + IV	82	18	64	

^a^Kruskal–Wallis *H-*test. KPS, Karnofsky performance status.

### Survival analysis

The clinical prognostic factors for survival of glioma patients were determined by univariate survival analyses using the Kaplan-Meier method and a log-rank test. The 138 patients whose samples were used for immunohistochemical staining were assessed for 5 years after surgery, and the mean length of postoperative follow-up for these patients was 34 months. Eighty-five patients (61.6 %) died due to tumor-related causes during this period. The Kaplan-Meier analysis indicated that p28GANK expression was related to a poor postoperative survival rate (log-rank test, *P* < 0.001) (Figure 1B). In addition, both patients with gradeI + II (*P* = 0.002) (Figure 1j) and those with grade III + IV (*P* = 0.001) (Figure 1k) with higher p28GANK expression correlated with poor patient prognosis.

Univariate analysis showed that, in addition to p28GANK expression, advanced WHO grade (*P* = 0.001) and high KPS (*P* < 0.001) were correlated with poor patient survival (Table 2). Multivariate analysis indicated that KPS (*P* = 0.003) and p28GANK expression (*P* = 0.003) were two independent prognostic factors (Table 2).

**Table 2:** Univariate and multivariate analysis of different prognostic factors correlated with patient survival

**Univariate analysis**^**a**^			**Multivariate analysis**^**b**^		
**Parameter**	**MST, months**	** *P* -value**	**Hazard ratio**	**95% CI**	** *P* -value**
Age, years		0.55	1.12	0.82, 1.97	0.29
< 42	34 (3.1)				
> 42	38 (6.8)				
Gender		0.39	0.08	0.61, 1.45	0.78
Men	37.2 (2.3)				
Women	40.2 (2.5)				
KPS, score		< 0.001	8.86	1.29, 3.49	0.003
< 70	—				
> 70	21 (2.1)				
Grade		0.001	3.43	0.97, 2.61	0.06
I + II	—				
III + IV	29 (3.1)				
p28GANK		< 0.001	8.61	1.37, 4.83	0.003
Negative	—				
Postivie	28 (1.58)				

MST: median survival time. ^a^Log-rank test. ^b^Cox regression model.

### Knockdown of p28GANK inhibited glioma cell growth

**Figure 2:**
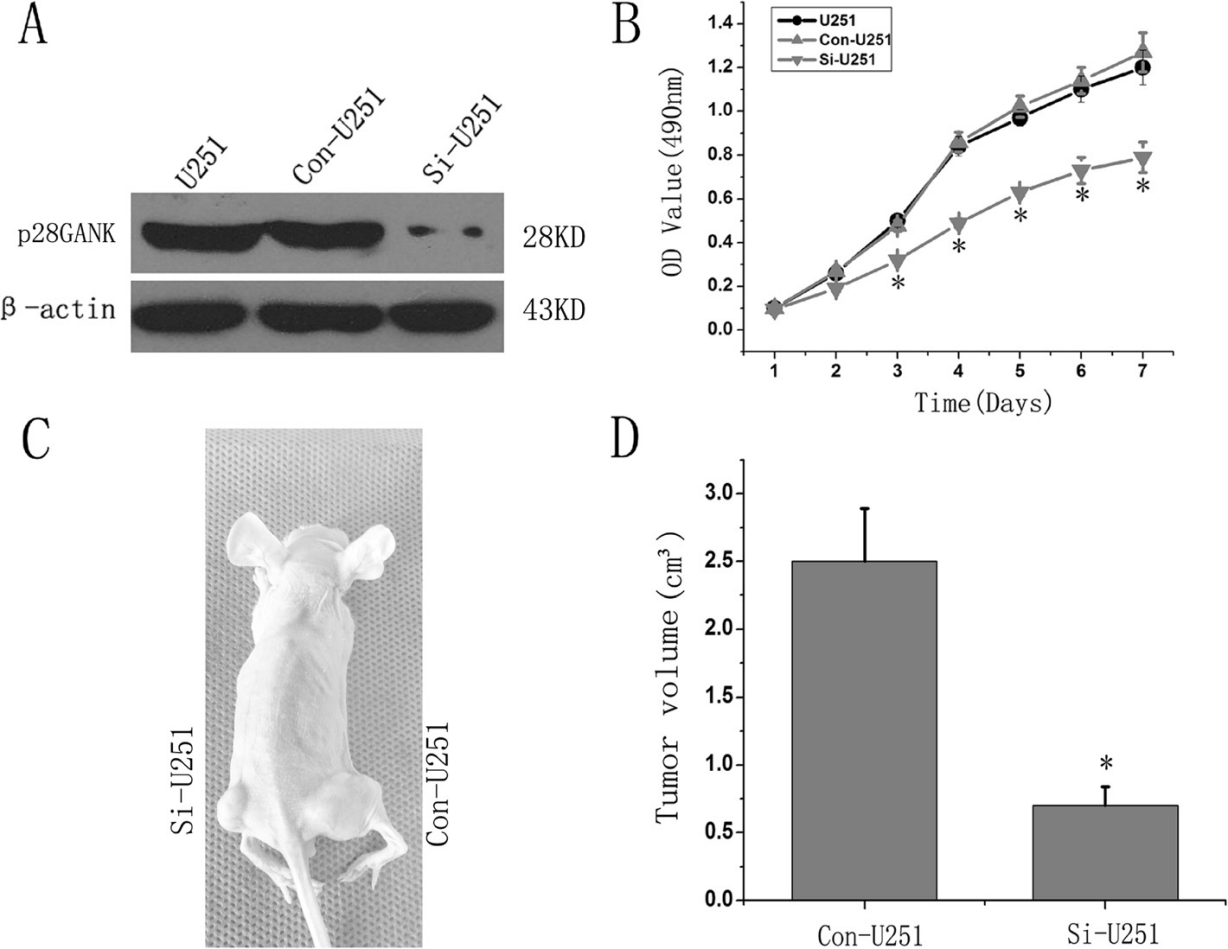
**Inhibition of p28GANK expression represses the proliferation of U251 cell *in vitro* and *in vivo.* ** (**A**) The expression of p28GANK in Si-U251 cells was markedly lower compared with the controls (Con-251 and U251) on Western blot analysis. (**B**): Growth curves of U251, Con-U251 and Si-U251 cells by MTT assays. The results presented were the mean of three replicates. (**C**) Comparison of glioma cell tumorigenicity in mice. The left flank was injected with Si-U251 cells, and the right flank was injected with Con-U251 cells. (**D**) Tumor volumes formed by si-U251 were significantly smaller than Con-U251 cells. *P* < 0.05.

Because the expression of p28GANK in gliomas markedly correlated with poor clinical outcome, we postulated that inhibition of the expression of p28GANK could repress the growth of glioma cells. To investigate the influence of p28GANK on the growth and proliferation of glioma cells, lentivirus-mediated siRNA targeting p28GANK was transfected into U251 cells. Western blot analysis confirmed that the endogenous expression of p28GANK was down-regulated in Si-U251 cells compared with the controls (Con-U251 and U251 cells; Figure 2A). An MTT assay was performed to detect cell-growth ability *in vitro.* The results showed that the growth of Si-U251 cells was much slower than that of the controls, and significant inhibitor effects were observed from the third day onward (Figure 2B), revealing that the inhibitory effect of p28GANK-siRNA on proliferation was likely caused by the knockdown of p28GANK expression. To further study the influence of p28GANK downregulation on tumor formation *in vivo*, BALB/c nu/nu mice were used for subcutaneous tumor formation assays. A month after cell injection, tumors in the left flanks (injected with Si-US251 cells) were significantly smaller than those in the right flanks (injected with Co-US251 cells) (*P* < 0.01) (Figure 2, C and D). Taken together, our present study suggested that the downregulation of p28GANK significantly inhibits glioma cell growth and proliferation both *in vitro* and *in vivo.*

## Discussion

Human gliomas are one of the most aggressive and malignant types of cancers, and present a great challenge for medical workers due to the lack of a desirable molecular marker that could improve patients’ prognostic classification. Therefore, additional efforts are essential for the identification of new markers for early diagnosis and therapeutic targets. P28GANK is over-expressed in human HCC, ESCC: esophageal squamous cell carcinoma and colorectal cancer and promotes cell-cycle progression [13-15], which is also associated with multi-drug resistance in gastric cancer and HCC cells [18,19], indicating that p28GANK might be a critical oncogene. However, the expression of p28GANK and its significance in human glioma are still unknown.

In the present study, our novel findings showed that the expression of p28GANK was markedly higher in gliomas than in matched para-cancerous tissues. Positive p28GANK expression was detected in 68.9% (95/138) of the glioma tissues, and high expression of p28GANK significantly correlated with KPS, WHO grade and poor overall survival, but not with patient age or gender. Univariate analysis showed that, in addition to p28GANK expression, WHO grade and KPS also correlated with patient survival, and multivariate analysis suggested that KPS and p28GANK expression were two independent prognostic factors for patients with gliomas. These findings were consistent with the roles of p28GANK in HCC, ESCC and pancreatic cancer, indicating that p28GANK might be an important prognostic marker for glioma patients, and might play a significant role in the pathogenesis of gliomas.

P28GANK can bind to MDM2, and increase the activities of MDM2 on p53 polyubiquitylation [20]. As a negative regulator of p53 and Rb [21,22], p28GANK plays physiological and physiopathological roles in cell-cycle progression by increasing the phosphorylation and degradation of Rb protein, and binding with CDK4 [23]. Our present study showed that over-expression of p28GANK correlated with poor clinical outcome of glioma patients. Thus, we determined the effects of p28GANK on growth and proliferation of glioma cells. Lentivirus-mediated siRNA can highly perturb targeted gene expression with high specificity and low toxicity, providing a powerful tool for the study of interested proteins [24]. To investigate the influence of p28GANK on the growth and proliferation of glioma cells, we constructed a lentivirus-mediated siRNA that targeted p28GANK and transfected into the U251cell line, which highly expressed p28GANK. The results showed that p28GANK expression was significantly downregulated in U251 cells transfected with the siRNA, and knockdown of p28GANK expression inhibited the growth ability of the glioma cell line U251 *in vitro*. This was further supported by the result that the tumors formed by siRNA-transfected U215 cells were markedly smaller compared with controls in nude mice, indicating that p28GANK gene silencing decreases the malignant growth of U251 cells both *in vitro* and *in vivo.* These findings might be elucidated by the report that downregulation of p28GANK induces an accumulation of cells in the G1/S phase [25]. Therefore, glioma patients may benefit from a targeted inhibition of p28GANK expression. However, the molecular mechanisms of p28GANK in the malignant progression of gliomas need to be studied further.

## Conclusions

In summary, this study provides novel findings that the increased expression of p28GANK correlates with poor clinical outcomes for patients with gliomas. A decreased expression of p28GANK effectively represses cell proliferation both *in vivo* and *in vitro*, indicating that p28GANK may be a potential therapeutic target for glioma treatment.

## Abbreviations

ANOVA, analysis of variance; DMEM, Dubecco modified eagle medium; DMSO, dimethyl sulfoxide; ELISA, enzyme-linked immunosorbent assay; FBS, fetal bovine serum; GFP, green fluorescent protein; HCC, hepatocellular carcinoma; KPS, Karnofsky performance status; PBS, phosphate-buffered saline; WHO, World Health Organisation.

## Competing interests

The authors declare that they have no competing interests.

## Authors’ contributions

YY carried out the molecular genetic studies, and drafted the manuscript; Chunli Zhang carried out IHC and the analysis of IHC; YG carried out the animal study; ZF carried out the experiment design; XL participated in IHC; YZ carried out the experiment design; BH participated in the sequence alignment. LL carried out the experiment design, and drafted the manuscript. WL carried out the experiment design, and drafted the manuscript. All authors read and approved the final manuscript.
